# Association between Tumor Vasculogenic Mimicry and the Poor Prognosis of Gastric Cancer in China: An Updated Systematic Review and Meta-Analysis

**DOI:** 10.1155/2016/2408645

**Published:** 2016-10-12

**Authors:** Qiujun Guo, Yuan Yuan, Zhichao Jin, Tao Xu, Yebo Gao, Huamin Wei, Conghuang Li, Wei Hou, Baojin Hua

**Affiliations:** ^1^Department of Oncology, Guang'anmen Hospital, China Academy of Chinese Medicine Sciences, No. 5 Beixiange, Xicheng District, Beijing 100053, China; ^2^Beijing University of Chinese Medicine, No. 11 North Third Ring Road East, Chaoyang District, Beijing 100029, China

## Abstract

*Background*. Vasculogenic mimicry can promote tumor growth and metastasis. This article is aimed at conducting a systematic meta-analysis to explore the clinicopathological and prognostic significance of vasculogenic mimicry and gastric cancer.* Methods*. We searched Pubmed, EMBASE, Cochrane Library, China National Knowledge Infrastructure, and the VIP and Wanfang Database for eligible studies. We manually searched for printed journals and relevant textbooks. Subgroups analyses were performed based on the region, manuscript quality, methods of vasculogenic mimicry identification, pathology, and number of patients.* Results*. Nine studies with 997 patients were included in this meta-analysis. A significant association was observed between vasculogenic mimicry-positive patients and those with gastric cancer with poor overall survival (hazard ratio = 2.24, 95% confidence interval: 1.45–3.47), poor pathological grading, high tumor node metastasis clinical stage, lymph node metastasis, deep tumor invasion, and distant metastasis.* Conclusions*. Vasculogenic mimicry is associated with a poor prognosis in patients with gastric cancer in China. Clinical studies with large samples are needed worldwide and standardized protocols should be adopted in the future to achieve a better understanding of the relationship between gastric cancer and vasculogenic mimicry.

## 1. Introduction

Gastric cancer (GC) is a leading cause of death worldwide, accounting for 0.7 million deaths and nearly 1 million newly diagnosed cases in 2012 [1, 2]. There is a high incidence of and high mortality from GC in China, making up more than one-third of the world population [3]. Although a variety of treatments such as surgery, chemotherapy, radiation therapy, and targeted therapy have an effect on GC, the 5-year survival rate still remains low, especially in cases of recurrence and metastasis [4].

Vasculogenic mimicry (VM) was first found in melanoma in 1999, and it indicated that tumor cells can directly generate vascular channels that facilitate tumor perfusion independent of tumor angiogenesis by vascular endothelial cells [5]. Based on the aforementioned features, VM can be distinguished by using the immunohistochemical or histochemical double staining, as VM is recognized as periodic acid-Schiff (PAS) positive and CD31 or CD34 (endothelial markers) negative, whereas classic blood vessels are double positive for PAS and endothelial markers.

VM can promote tumor growth and metastasis, and it is closely related to tumor neovascularization and cancer stem-like cells, which are considered hallmarks of cancer, and they are associated with tumor invasion and drug resistance [6]. Recent studies have shown that VM is associated with a poor prognosis in human tumors [7–11]. A meta-analysis [12] indicated no significant relationship between VM and GC. It only included two eligible studies on GC with 257 patients and minimal valuable clinical evidence. However, a recent study indicated that VM may play an important role in the evolution of GC [13]. Based on the aforementioned controversy, we conducted this meta-analysis to evaluate the relationship between VM and the prognosis and clinicopathological features of patients with GC. This study was conducted according to the PRISMA guidelines (see S6 file in Supplementary Material available online at http://dx.doi.org/10.1155/2016/2408645) [14, 15].

## 2. Materials and Methods

### 2.1. Identification of Eligible Studies

We searched Pubmed, EMBASE, Cochrane Library, China National Knowledge Infrastructure, and the VIP and Wanfang Database for eligible studies without using language limits. The search period was from January 1999 to October 2015. The search terms were as follows: “vasculogenic mimicry or tumour cell-lined vessels” and “gastric or stomach”. We used both Medical Subject Heading terms and free-text words to increase the sensitivity of the search.

In addition to electronic databases, printed journals and relevant textbooks were manually searched in the libraries of Beijing University of Chinese Medicine, Peking Union Medical College, and Guang'anmen Hospital. Specialized experts in particular fields were consulted for necessary supplements as well.

Inclusion criteria were as follows: (1) studies on patients with a histological diagnosis of GC; (2) articles on those with VM-positive primary tumor tissues assessed by the immunohistochemical or histochemical double staining method; and (3) studies aiming to assess the relationship between VM and at least one of the following outcome variables and clinicopathological features: overall survival (OS) time, tumor node metastasis clinical stage, lymph node metastasis, poor pathological grade, blood metastasis, and depth of tumor invasion.

Exclusion criteria were as follows: (1) reviews and single case reports; (2) studies referring to VM but not to humans with VM with gastric cancer; and (3) studies that lacked outcome variables and clinicopathological features.

### 2.2. Data Extraction and Management

Two independent reviewers (Yebo Gao and Zhichao Jin) extracted the data by using a standardized collection form according to the aforementioned inclusion and exclusion criteria of eligible studies. We recorded the details of eligible studies, including the first author, patients' region, publication year, pathological type, VM assay methods, total cases, clinicopathological features, and outcomes. If there were discrepancies between the two reviewers, a final consensus was reached after discussion with the other author (Yuan Yuan). The hazard ratio (HR) was calculated from the Kaplan-Meier survival curve and 5-year survival outcome events as reported by Tierney et al. [16].

### 2.3. Methodological Assessment

The methodological assessment of eligible studies was conducted by using the quality scale for biological prognostic factors (S5 file), which was reported previously [17] by two specialists (Huamin Wei and Tao Xu) who are experienced in clinical and basic experiments. Disagreements were discussed with another specialist (Conghuang Li).

### 2.4. Statistical Analyses

Statistical analyses were performed using Review Manager (RevMan) 5.3.5 software (Cochrane Community, London, United Kingdom) and STATA 14 software (STATA Corp., College Station, TX). Dichotomous data of the clinicopathological features were pooled using odds ratios (ORs) with 95% confidence intervals (CIs). HRs were pooled as inverse variance data with 95% CI. *P* < 0.05 was considered to indicate a statistically significant difference. An observed HR or OR > 1 implied a worse prognosis for the group with VM positivity, and it was considered statistically significant if the values of 95% confidence intervals did not overlap the value “1.” The heterogeneity of the included studies was evaluated by the *χ* ^2^ and *I* ^2^ tests, and *P* < 0.10 or *I* ^2^ > 50% was defined as heterogeneous. The fixed-effect model was used for merging the homogeneous data, and the random-effects model was suitable for merging the heterogeneous data as previously reported [26]. Publication bias was evaluated by Egger's test (STATA 14) with *P* < 0.05 indicating potential bias. The sensitivity analysis was evaluated by reanalyzing the data using different statistical approaches.

## 3. Results

### 3.1. Characteristics of the Included Studies

**Figure 1:**
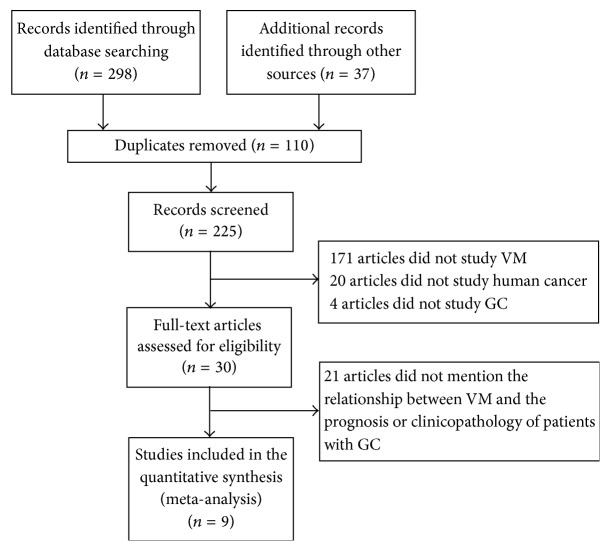
Flow diagram of the literature search process. VM, vasculogenic mimicry; GC, gastric cancer.

Three hundred thirty-five studies, including 37 additional records identified through other sources such as postgraduate dissertations and conference articles, were initially found by using the aforementioned search strategy. One hundred ten duplicate studies were removed, 171 were excluded because they did not study VM, 20 were excluded because they did not study human cancer, and 4 were excluded because they did not study GC. After reading the full text, 21 studies were excluded because they did not mention the relationship between VM and the prognosis or clinicopathology of patients with GC. Finally, 9 studies with 997 patients were included (Figure 1 and Table 1).

Six articles studied the HR between the OS and VM. HRs were calculated using the method previously reported by Tierney et al. [16] (S4 file Table 1). The methodological assessment of eligible studies was conducted as described in Table 2.

### 3.2. Results of the Meta-Analysis

#### 3.2.1. VM Positivity Indicated a Poor Prognosis in Patients with GC

**Figure 2:**
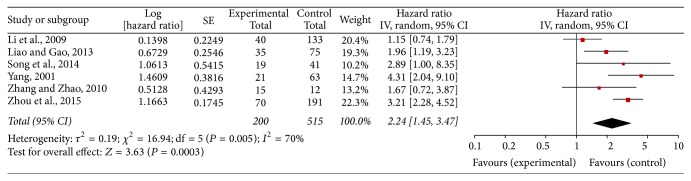
Forest plot of hazard ratios (HRs) in the random-effect model. The HR of overall survival of vasculogenic mimicry- (VM-) positive cancer patients was compared with VM-negative cancer patients. Each individual study is represented by the red square, and the pooled datasets are indicated by the diamond, representing the 95% confidence interval (CI) of each study. An HR > 1 implied a worse survival for the cancer patients. The size of each study represents the weighting factor (1/standard error [SE]) assigned to it.

Six studies reported the OS of patients with GC with VM positivity for 715 patients. A significant association was observed between VM positivity and OS. This result suggested that VM positivity may represent a poor prognostic factor for patients with GC (random-effect model: HR = 2.24, 95% CI: 1.45–3.47) (Figure 2). An insignificant heterogeneity was detected among the studies (chi^2^ = 16.94, df = 5, *P* = 0.005, and *I* ^2^ = 70%). The sensitive analysis was performed by using the fixed-effects model (HR = 2.25, 95% CI: 1.82–2.79) (S1 file Figure  1.5). The results of the two models were comparable.

### 3.3. Subgroup Analyses

Due to the presence of heterogeneity, subgroups analyses were performed based on the region, methods of VM identification, pathology, and number of patients (Table 3 and S1 file Figures  1.1–1.5).

We detected a significant association between VM positivity and the OS of patients with GC in inland regions (HR = 2.90, 95% CI: 2.23–3.78) but not in coastal regions (HR = 1.38, 95% CI: 0.96–1.99). VM positivity was significantly associated with the OS of patients with adenocarcinoma (HR = 2.36, 95% CI: 1.44–3.86) but was not significantly associated with the OS of patients with sarcoma (HR = 1.67, 95% CI: 0.72–3.87). The association between VM positivity and the OS of patients was present in studies with more than 100 or fewer than 100 subjects (HR = 1.96, 95% CI: 104–3.66; HR = 2.85, 95% CI: 1.74–4.67). In addition, analysis of the subgroups using different VM detection methods showed a poor OS in the PAS-positive and CD34-negative staining subgroup (HR 2.60, 95% CI: 1.61–4.19) and PAS-positive and CD31-negative staining subgroup (HR 2.10, 95% CI: 1.07–4.10). Significant heterogeneity existed among the studies with the methods of VM identification, pathological type, and sample size subgroups; however, there was no significant heterogeneity in the subgroups for the study region.

Furthermore, results of the sensitivity analyses showed that changing the study effect model did not change the results of the pooled analyses of OS.

### 3.4. Associations between VM Positivity and the Clinicopathological Characteristics of Patients with GC

The prognostic significance of VM positivity in the TNM clinical stage was evaluated in 6 studies [13, 18–20, 22, 24] with 789 patients. The results showed that VM positivity can lead to a high TNM clinical stage (III or IV clinical stage) in patients with GC (random-effects model: OR = 3.12, 95% CI: 1.52–6.42) with a significant heterogeneity. In the analysis of 7 studies [13, 18–20, 22–24] with 910 patients, VM positivity was significantly associated with lymph node metastasis in patients with GC (fixed-effect model: OR = 2.82, 95% CI: 2.04–3.92). We evaluated the relationship between VM positivity and the pathological differentiation in 7 studies [13, 18–20, 22–24] with 910 patients. The results showed that VM positivity can induce poorer pathological differentiation (fixed-effect model: OR = 3.64, 95% CI: 2.53–5.24). The prognostic significance of VM positivity in patients with blood metastasis was analyzed in 3 studies [19, 22, 24] with 331 patients. We observed a significant relationship between VM positivity and blood metastasis in patients with GC (fixed-effect model: OR = 4.34, 95% CI: 1.57–11.96). The depth of invasion in patients with GC tended to correlate with VM positivity in the evaluation of 2 studies [13, 22] with 335 patients (fixed-effect model: OR = 2.95, 95% CI: 1.63–5.35). There was no significant heterogeneity between studies on lymph node metastasis, pathological differentiation, blood metastasis, and the depth of invasion. The aforementioned results are presented in Table 4 (Figures  S2.1–2.5 and S4 Table 1).

### 3.5. Publication Bias

The publication bias was assessed by Egger's test, and the results demonstrated no obvious publication bias in our meta-analysis (Figures  S3.1–3.5).

## 4. Discussion

VM is found in many kinds of tumors, and it is regarded as a poor prognostic marker in sarcomas [27], melanomas [28], hepatocellular carcinomas [29], laryngeal squamous cell carcinomas [30], colorectal cancers [31], gallbladder carcinomas [32], non-small cell lung cancers [33], and osteosarcomas [34]. Although the molecular mechanisms of VM are not fully understood, a study indicated that VM formation was associated with cancer stem cells that improved transendothelial migration in VM-forming cells [35]. Besides, VE-cadherin overexpression and metalloproteinases (MMPs), via their cleavage of laminin, promote adherence of the VM channel wall to tumor cells [36, 37]. Multiple signaling pathways contribute to VM formation. It was shown that vascular endothelial growth factor- (VEGF-) a-EphA2-MMPs were the main pathway for VM formation, and VEGF-a appeared to play an important role in the formation of VM [38]. The VEGF and phosphoinositide 3-kinase/AKT pathway also exert positive feedback regulation in the process of VM formation [39]. EphA2 is a protein tyrosine kinase receptor commonly expressed in epithelial cells [40], and it contributes to VM formation by mediating the EphA2/FAK/Paxillin pathway[41–44]. In the Wnt/*β*-catenin pathway, hypoxic conditions and microRNAs also take part in VM formation [45–47].

A previous study with insufficient clinical research indicated that VM was not closely associated with the prognosis of patients with GC [12]. However, our meta-analysis showed that VM was significantly associated with the short OS, poor pathological grading, high TNM clinical stage, lymph node metastasis, deep tumor invasion, and distant metastasis of patients with GC.

The heterogeneity of the included studies drew our attention. To determine the studies with heterogeneity, we applied the random-effects model and fixed-effect model and performed sensitivity analysis. The subgroup analysis showed significant heterogeneity for the methods of VM identification subgroups and those with sample size more than 100 subgroup and adenocarcinoma pathological type subgroup, indicating that the following factors may account for the heterogeneity. Firstly, the quality of the included studies varied, as some studies may be comparatively not well designed. Secondly, VM was detected in different ways (e.g., PAS-positive and CD31- or CD34-negative staining). Differences in the quality of the antibodies, laboratory reagents, conditions, and pathological evaluation standards may have resulted in this heterogeneity. Furthermore, according to our subgroup analysis, we found that a significant association between VM positivity and the OS was detected for gastric adenocarcinoma patients in inland regions in China.

There were some limitations in our study. First, all studies included in our evaluation were conducted in China; hence, the conclusion can only be carefully applied to China or East Asia, not worldwide. Second, there was some heterogeneity among the eligible studies, and their global scores were diverse. These limitations may have influenced our evaluation, but the same results were reached in the sensitivity analysis via different testing models.

In conclusion, VM was associated with the poor prognosis of patients with GC in China, especially for those gastric adenocarcinoma patients in inland regions; however, drugs targeting VM should be studied and used in GC treatment. Furthermore, clinical studies with large samples are needed to evaluate the relationship between GC and VM worldwide, and standardized protocols should be adopted in future studies.

## Supplementary Material

The information of supplementary materials are as follows: S1 file. Results of subgroup analysis of the included studies and analysis of hazard ratios (HRs) in the random-effect model; S2 file. Meta-analysis of VM and clinical and pathologic features in GC patients; S3 file. Egger tests; S4 file. Data of eligible studies; S5 file. Quality scale for biological prognostic factors; S6 file. PRISMA 2009 Checklist .

## Figures and Tables

**Table 1:** Characteristics of the included studies.

Study	Year	Region	Sample size (*n*)	Number of VM-positive patients (%)	Pathological type	Clinical stage	Methods of VM identification	Preoperative treatment	Clinicopathological features	Outcome measures	Survival analysis
Chen [18]	2009	Hunan, China	87	21 (24.1)	Adenocarcinoma	I–IV	PAS^+^CD34^−^	No	Pathological grade Blood metastasis TNM clinical stage	—	—

Li et al. [19]	2009	Tianjin, China	173	40 (23.1)	Adenocarcinoma	I–IV	PAS^+^CD31^−^	No	Pathological grade Lymph node metastasis Blood metastasis TNM clinical stage	OS	Multivariate

Liao and Gao [20]	2013	Chongqing, China	110	35 (31.8)	Adenocarcinoma	I–IV	PAS^+^CD34^−^	Unclear	Pathological grade Lymph node metastasis TNM clinical stage	OS	Univariate

Song et al. [21]	2014	Shandong, China	60	19 (31.7)	Adenocarcinoma	I–IV	PAS^+^CD31^−^	No	—	OS	Multivariate

Su et al. [22]	2015	Hebei, China	74	22 (29.7)	Adenocarcinoma	I–IV	PAS^+^CD34^−^	No	Pathological grade Degree of invasion Intravascular cancer embolus Lymph node metastasis Blood metastasis TNM clinical stage	—	—

Wang et al. [23]	2010	Anhui, China	121	44 (36.4)	Adenocarcinoma	Unclear	PAS^+^CD34^−^	No	Pathological grade Lymph node metastasis	—	—

Yang [24]	2001	Hunan, China	84	21 (25.0)	Adenocarcinoma	I–IV	PAS^+^CD31^−^	No	Pathological grade Lymph node metastasis TNM clinical stage	OS	Multivariate

Zhang and Zhao [25]	2010	Shandong, China	27	15 (55.6)	Sarcoma	I–III	PAS^+^CD31^−^	Unclear	—	OS	Univariate

Zhou et al. [13]	2015	Anhui, China	261	70 (26.8)	Adenocarcinoma	I–IV	PAS^+^CD34^−^	No	Pathological grade Lymph node metastasis TNM clinical stage Degree of invasion	OS	Multivariate

**Table 2:** Quality assessment of the included studies.

Study	Scientific design	Laboratory methodology	Generalizability	Results analysis	Global score (%)
Chen [18]	9	12	8	0	73
Li et al. [19]	9	8	10	6	83
Liao and Gao [20]	9	5	5	4	58
Song et al. [21]	9	8	7	5	73
Su et al. [22]	9	7	8	0	60
Wang et al. [23]	7	5	8	0	50
Yang [24]	9	10	8	6	83
Zhang and Zhao [25]	8	4	3	5	50
Zhou et al. [13]	9	6	8	5	70

**Table 3:** Results of the subgroup analysis of the included studies.

Study subgroups	Number of studies	Number of patients	Pooled HR [95% CI]				Heterogeneity	
			Fixed	*P* value	Random	*P* value	*I* ^2^ (%)	*P* value
Study region								
Coastal region	3	260	1.38 [0.96, 1.99]	0.09	1.48 [0.92, 2.39]	0.11	26	0.26
Inland	3	455	2.90 [2.23, 3.78]	<0.00001	2.89 [1.94, 4.30]	<0.00001	47	0.15
Methods of VM identification								
PAS^+^CD34^−^	2	371	2.74 [2.07, 3.64]	<0.00001	2.60 [1.61, 4.19]	<0.0001	61	0.11
PAS^+^CD31^−^	4	344	1.72 [1.24, 2.39]	0.001	2.10 [1.07, 4.10]	0.03	70	0.02
Pathological type								
Adenocarcinoma	5	688	2.30 [1.84, 2.87]	<0.00001	2.36 [1.44, 3.86]	0.0007	76	0.002
Sarcoma	1	27	1.67 [0.72, 3.87]	0.03	1.67 [0.72, 3.87]	0.23	Not applicable	
Sample size (*n*)								
>100	3	544	2.13 [1.68, 2.70]	<0.00001	1.96 [1.04, 3.66]	0.04	85	0.001
<100	3	171	2.85 [1.74, 4.67]	<0.0001	2.81 [1.57, 5.05]	0.0005	27	0.26

VM, vasculogenic mimicry; OR, odds ratio; CI, confidence interval; HR, hazard ratio; PAS, periodic acid-Schiff.

**Table 4:** Meta-analysis of VM and the clinical and pathological features of patients with GC.

Clinical and pathological features	Number of studies	Number of patients	Pooled OR [95% CI]				Heterogeneity	
			Fixed	*P* value	Random	*P* value	*I* ^2^ (%)	*P* value
III/IV clinical stage	6 [13, 18–20, 22, 24]	789	3.35 [2.31, 4.86]	<0.00001	3.12 [1.52, 6.42]	<0.0001	65	0.01
Lymph node metastasis	7 [13, 18–20, 22–24]	910	2.82 [2.04, 3.92]	<0.00001	2.84 [1.95, 4.14]	<0.00001	20	0.28
Poor differentiation	7 [13, 18–20, 22–24]	910	3.64 [2.53, 5.24]	<0.00001	3.92 [2.33, 6.59]	<0.00001	35	0.16
Blood metastasis	3 [19, 22, 24]	331	3.79 [2.14, 6.71]	<0.00001	4.34 [1.57, 11.96]	0.005	54	0.11
T3/4 invasion	2 [13, 22]	335	2.95 [1.63, 5.35]	0.0003	3.06 [1.29, 7.27]	0.01	17	0.27

VM, vasculogenic mimicry; GC, gastric cancer; OR, odds ratio; CI, confidence interval.
