# Supplementary material for: Association between Tumor Vasculogenic Mimicry and the Poor Prognosis of Gastric Cancer in China: An Updated Systematic Review and Meta-Analysis
Source: Biomed Res Int. 2016 Oct 12;2016:2408645. doi: 10.1155/2016/2408645 (PMC5080470; doi:10.1155/2016/2408645)
Supplement: Supplementary file 1 — The information of supplementary materials are as follows: S1 file. Results of subgroup analysis of the included studies and analysis of hazard ratios (HRs) in the random-effect model; S2 file. Meta-analysis of VM and clinical and pathologic features in GC patients; S3 file. Egger tests; S4 file. Data of eligible studies; S5 file. Quality scale for biological prognostic factors; S6 file. PRISMA 2009 Checklist . [file 2408645.f1.doc]

**S1 file. Results of subgroup analysis of the included studies and analysis of hazard ratios (HRs) in the random-effect model**


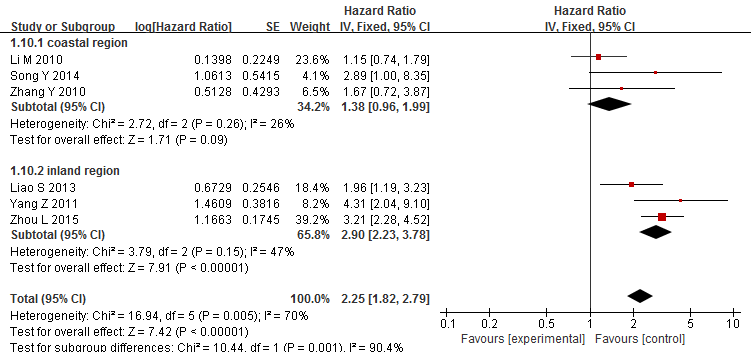


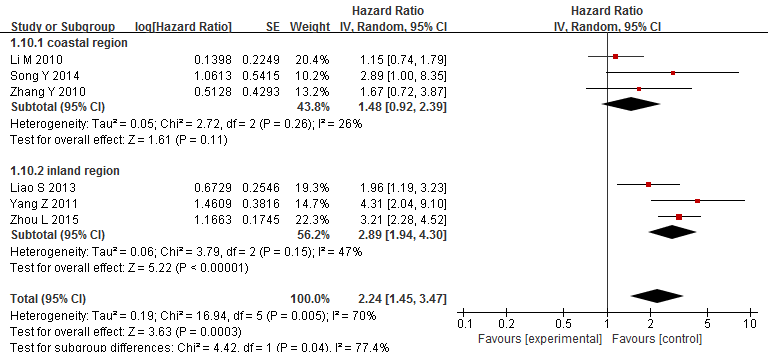


Figure 1.1: Forrest plot of hazard ratio in fix/random effect model of subgroup analysis of study regions (coastal region and inland region). The HR of OS of VM-positive cancer patients was compared with VM-negative cancer patients. Individual study is shown in the square with red color, and the pooled datasets were shown in the diamond, representing the 95% conﬁdence interval (CI) of all each study. HR > 1 implied a worse survival of the cancer patients. The size of each investigation represented the weighting factor (1/SE) assigned to the study.


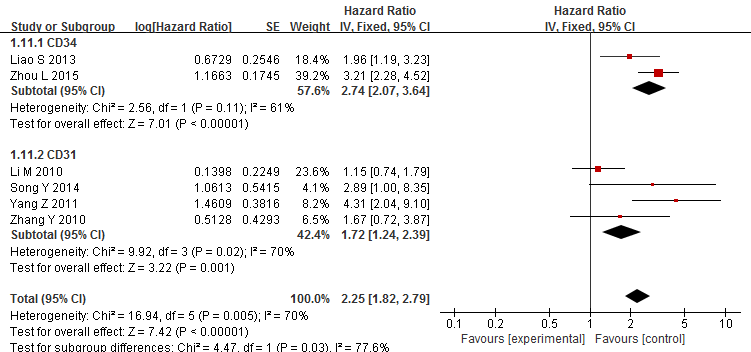


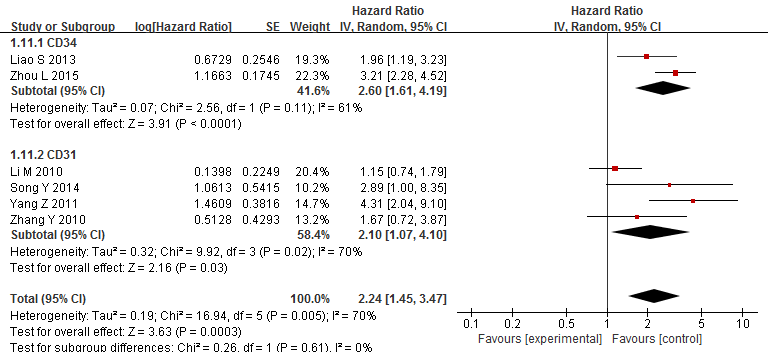


Figure 1.2: Forrest plot of hazard ratio in fix/random effect model of subgroup analysis of VM identification methods (PAS^+^CD34^-^ and PAS^+^CD31^-^). The HR of OS of VM-positive cancer patients was compared with VM-negative cancer patients. Individual study is shown in the square with red color, and the pooled datasets were shown in the diamond, representing the 95% conﬁdence interval (CI) of all each study. HR > 1 implied a worse survival of the cancer patients. The size of each investigation represented the weighting factor (1/SE) assigned to the study.


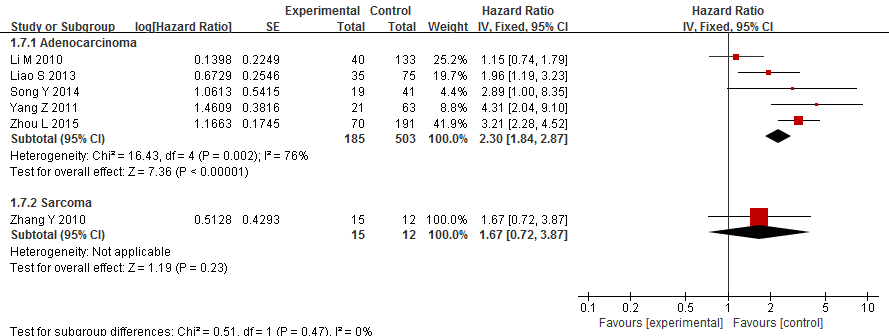


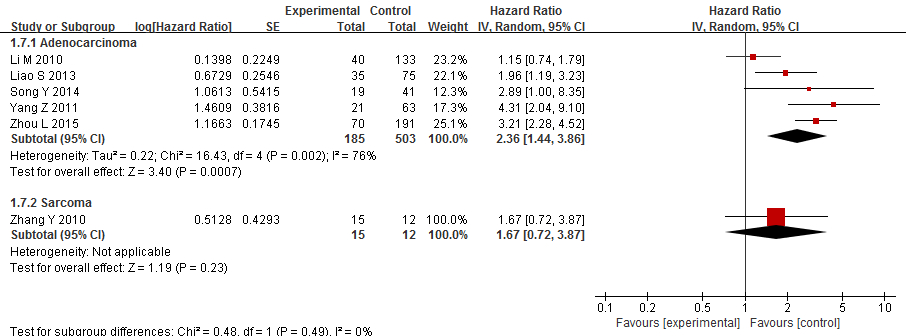


Figure 1.3: Forrest plot of hazard ratio in fix/random effect model of subgroup analysis of pathological types (adenocarcinoma and sarcoma). The HR of OS of VM-positive cancer patients was compared with VM-negative cancer patients. Individual study is shown in the square with red color, and the pooled datasets were shown in the diamond, representing the 95% conﬁdence interval (CI) of all each study. HR > 1 implied a worse survival of the cancer patients. The size of each investigation represented the weighting factor (1/SE) assigned to the study.


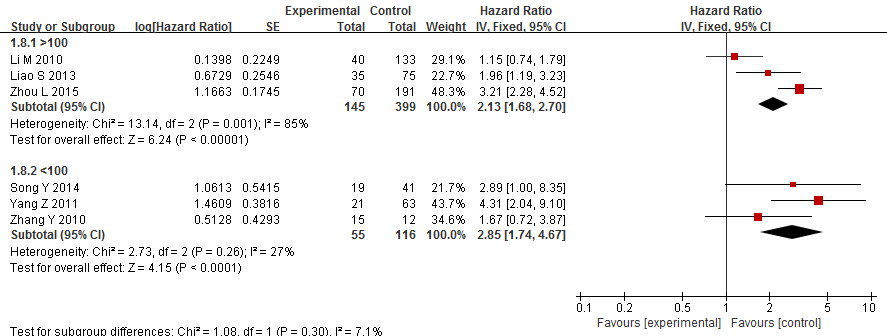


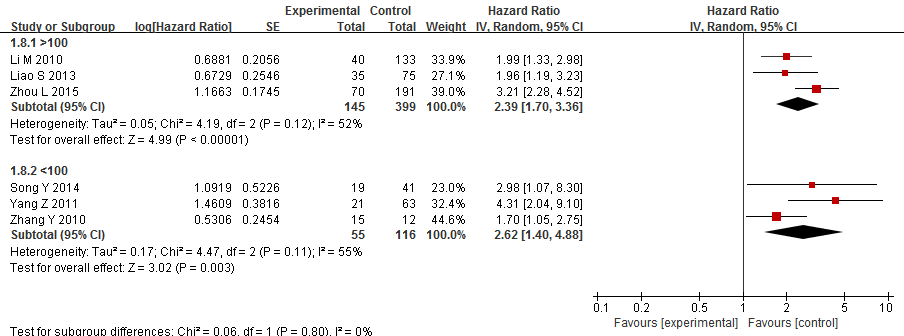


Figure 1.4: Forrest plot of hazard ratio in fix/random effect model of subgroup analysis of sample sizes (>100 and <100). The HR of OS of VM-positive cancer patients was compared with VM-negative cancer patients. Individual study is shown in the square with red color, and the pooled datasets were shown in the diamond, representing the 95% conﬁdence interval (CI) of all each study. HR > 1 implied a worse survival of the cancer patients. The size of each investigation represented the weighting factor (1/SE) assigned to the study.


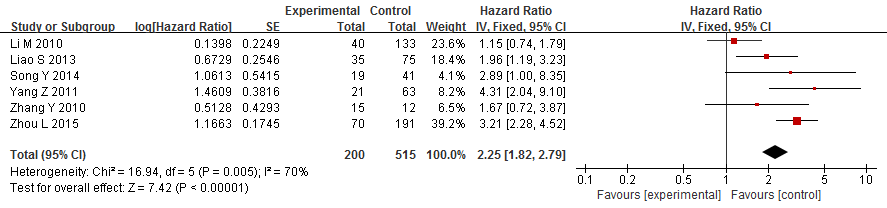


Fig 1.5: Forest plot of hazard ratios (HRs) in the fixed-effect model. The HR of overall survival of vasculogenic mimicry (VM)-positive cancer patients was compared with VM-negative cancer patients. Each individual study is represented by the red square, and the pooled datasets are indicated by the diamond, representing the 95% conﬁdence interval (CI) of each study. An HR >1 implied a worse survival of cancer patients. The size of each study represented the weighting factor (1/standard error [SE]) assigned to it.
