# Supplementary material for: Association between Tumor Vasculogenic Mimicry and the Poor Prognosis of Gastric Cancer in China: An Updated Systematic Review and Meta-Analysis
Source: Biomed Res Int. 2016 Oct 12;2016:2408645. doi: 10.1155/2016/2408645 (PMC5080470; doi:10.1155/2016/2408645)
Supplement: Supplementary file 2 [file 2408645.f2.doc]

**S2 file. Meta-analysis of VM and clinical and pathologic features in GC patients**


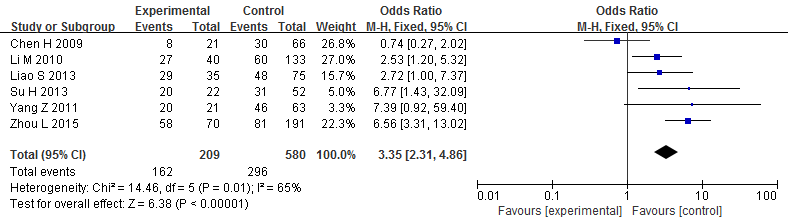


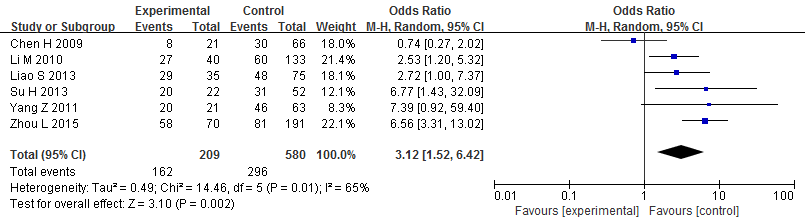


Figure 2.1. Forrest plot of hazard ratio in fix/random effect model of TNM clinic stages. The OR of III-IV TNM clinic stage of VM-positive cancer patients was compared with VM-negative cancer patients. Individual study is shown in the square with blue color, and the pooled datasets were shown in the diamond, representing the 95% conﬁdence interval (CI) of each study. OR > 1 implied a high TNM clinic stage of cancer patients. The size of each investigation represented the weighting factor (1/SE) assigned to the study.


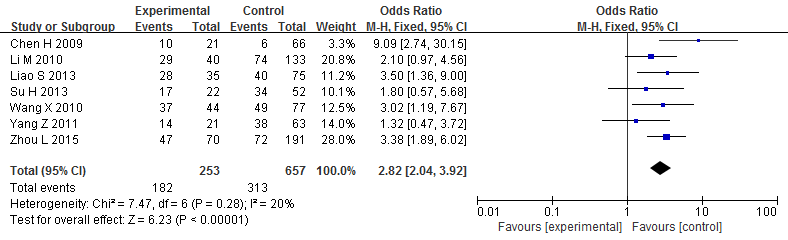


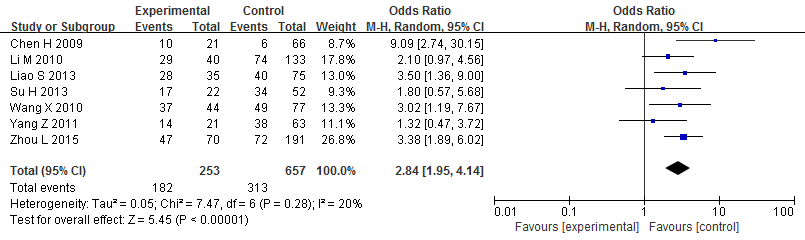


Figure 2.2. Forrest plot of hazard ratio in fix/random effect model of lymph nodes metastasis. The OR of lymph nodes metastasis of VM-positive cancer patients was compared with VM-negative cancer patients. Individual study is shown in the square with blue color, and the pooled datasets were shown in the diamond, representing the 95% conﬁdence interval (CI) of each study. OR > 1 implied more cancer patients with lymph nodes metastasis. The size of each investigation represented the weighting factor (1/SE) assigned to the study.


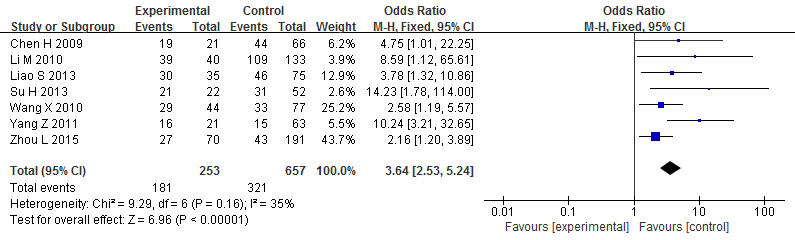


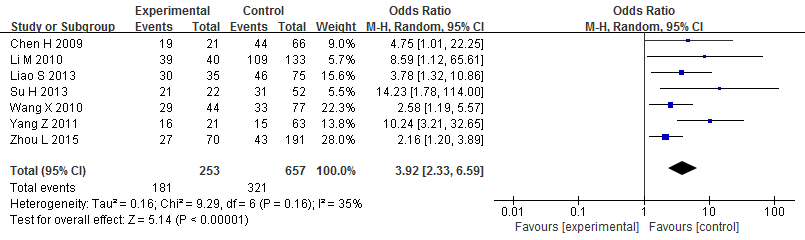


Figure 2.3. Forrest plot of hazard ratio in fix/random effect model of tumor pathological differentiation. The OR of the differentiation of VM-positive cancer patients was compared with VM-negative cancer patients. Individual study is shown in the square with blue color, and the pooled datasets were shown in the diamond, representing the 95% conﬁdence interval (CI) of each study. OR > 1 implied a poor differentiation of cancer patients. The size of each investigation represented the weighting factor (1/SE) assigned to the study.


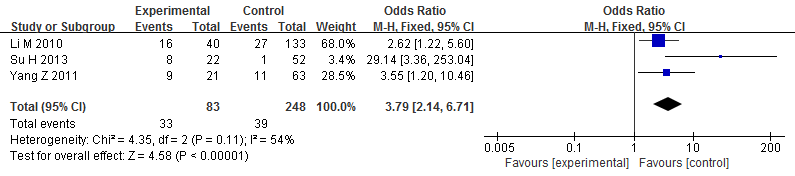


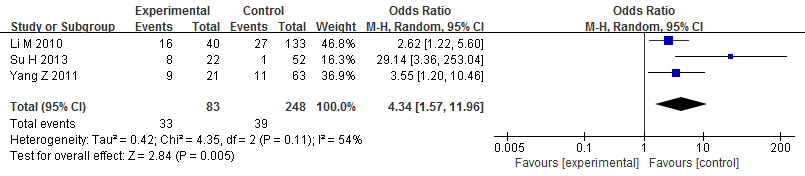


Figure 2.4. Forrest plot of hazard ratio in fix/random effect model of blood metastasis. The OR of the metastasis of VM-positive cancer patients was compared with VM-negative cancer patients. Individual study is shown in the square with blue color, and the pooled datasets were shown in the diamond, representing the 95% conﬁdence interval (CI) of each study. OR > 1 implied more cancer patients with distant metastasis. The size of each investigation represented the weighting factor (1/SE) assigned to the study.


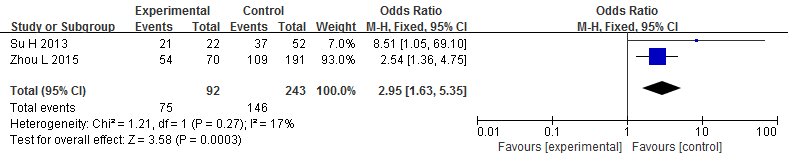


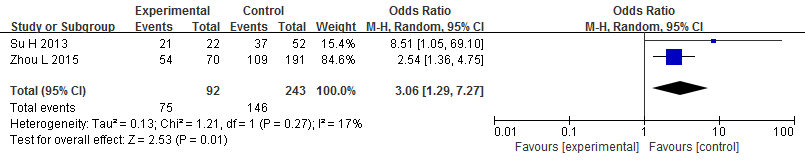


Figure 2.5. Forrest plot of hazard ratio in fix/random effect model of tumor T3/4 invasion. The OR of the invasion of VM-positive cancer patients was compared with VM-negative cancer patients. Individual study is shown in the square with blue color, and the pooled datasets were shown in the diamond, representing the 95% conﬁdence interval (CI) of each study. OR > 1 implied more cancer patients with T3/4 invasion. The size of each investigation represented the weighting factor (1/SE) assigned to the study.
