# Supplementary material for: Association between Tumor Vasculogenic Mimicry and the Poor Prognosis of Gastric Cancer in China: An Updated Systematic Review and Meta-Analysis
Source: Biomed Res Int. 2016 Oct 12;2016:2408645. doi: 10.1155/2016/2408645 (PMC5080470; doi:10.1155/2016/2408645)
Supplement: Supplementary file 3 [file 2408645.f3.doc]

**S3 file. Egger's tests of meta-analyses**

**3.1.Egger's test of HR**

Egger's test

------------------------------------------------------------------------------

Std_Eff | Coef. Std. Err. t P>|t| [95% Conf. Interval]

-------------+----------------------------------------------------------------

slope | .8143052 .6225274 1.31 0.261 -.9141079 2.542718

bias | -.0148549 2.325701 -0.01 0.995 -6.472037 6.442327

**3.2.Egger's test of III/IV clinical stage**

------------------------------------------------------------------------------

Std_Eff | Coef. Std. Err. t P>|t| [95% Conf. Interval]

-------------+----------------------------------------------------------------

slope | 1.785995 1.30575 1.37 0.243 -1.839347 5.411338

bias | -1.44297 2.679141 -0.54 0.619 -8.881458 5.995518

**3.3. Egger's test of lymph nodes metastasis**

------------------------------------------------------------------------------

Std_Eff | Coef. Std. Err. t P>|t| [95% Conf. Interval]

-------------+----------------------------------------------------------------

slope | 2.298461 .7351648 3.13 0.026 .40866 4.188263

bias | -3.477993 1.662123 -2.09 0.091 -7.750616 .7946294

**3.4.Egger's test of poor differentiation**

------------------------------------------------------------------------------

Std_Eff | Coef. Std. Err. t P>|t| [95% Conf. Interval]

-------------+----------------------------------------------------------------

slope | -2.18644 1.455276 -1.50 0.193 -5.927346 1.554465

bias | 5.284596 2.873773 1.84 0.125 -2.102673 12.67187

**3.5.Egger's test of blood metastasis**

------------------------------------------------------------------------------

Std_Eff | Coef. Std. Err. t P>|t| [95% Conf. Interval]

-------------+----------------------------------------------------------------

slope | 13.43366 8.010521 1.68 0.342 -88.34966 115.217

bias | -34.52738 15.15941 -2.28 0.263 -227.1459 158.0911
