# Supplementary material for: Association between Tumor Vasculogenic Mimicry and the Poor Prognosis of Gastric Cancer in China: An Updated Systematic Review and Meta-Analysis
Source: Biomed Res Int. 2016 Oct 12;2016:2408645. doi: 10.1155/2016/2408645 (PMC5080470; doi:10.1155/2016/2408645)
Supplement: Supplementary file 4 [file 2408645.f4.doc]

**S4 file.** **Data of eligible studies**

**Table 1 Clinicopathological data of eligible studies**

| Studies | VM(+)/(-) | No. of patients | III/IV clinical stage | Lymph node metastasis | Poor differentiation | Blood metastasis | T3/4 invasion |
| --- | --- | --- | --- | --- | --- | --- | --- |
| Chen H | VM (+) | 21 | 8 | 10 | 19 | - | - |
|  | VM (-) | 66 | 30 | 6 | 44 |  |  |
| Li M *et al* | VM (+) | 40 | 27 | 29 | 39 | 16 | - |
|  | VM (-) | 133 | 80 | 74 | 109 | 27 |  |
| Liao S *et al* | VM (+) | 35 | 29 | 28 | 30 | - | - |
|  | VM (-) | 75 | 48 | 40 | 46 |  |  |
| Song Y *et al* | VM (+) | 19 | - | - | - | - | - |
|  | VM (-) | 41 |  |  |  |  |  |
| Su H *et al* | VM (+) | 22 | 20 | 17 | 21 | 8 | 21 |
|  | VM (-) | 52 | 31 | 34 | 31 | 1 | 37 |
| Wang X *et al* | VM (+) | 44 | - | 37 | 29 | - | - |
|  | VM (-) | 77 |  | 49 | 33 |  |  |
| Yang Z | VM (+) | 21 | 20 | 14 | 16 | 9 | - |
|  | VM (-) | 63 | 46 | 38 | 15 | 11 |  |
| Zhang Y *et al* | VM (+) | 15 | - | - | - | - | - |
|  | VM (-) | 12 |  |  |  |  |  |
| Zhou L *et al* | VM (+) | 70 | 58 | 47 | 27 | - | 54 |
|  | VM (-) | 191 | 81 | 72 | 33 |  | 109 |
